# Permanent deterioration of fine motor skills after the resection of tumors in the supplementary motor area

**DOI:** 10.1007/s10143-024-02330-2

**Published:** 2024-03-14

**Authors:** Stefanie Maurer, Vicki M. Butenschoen, Anna Kelm, Severin Schramm, Axel Schröder, Bernhard Meyer, Sandro M. Krieg

**Affiliations:** 1 Department of Neurosurgery, School of Medicine, Klinikum rechts der Isar, Technical University of Munich, Ismaninger Str. 22, 81675 Munich, Germany; 2 Department of Neurosurgery, Goethe University Hospital, Frankfurt, Germany; 3 Department of Diagnostic and Interventional Neuroradiology, School of Medicine, Klinikum rechts der Isar, Technical University of Munich, Munich, Germany; 4 Department of Neurosurgery, University Hospital Heidelberg, Heidelberg, Germany

**Keywords:** Supplementary motor area, Fine motor skills, Jebsen-Taylor Hand Function Test, Nine-Hole Peg Test, Navigated transcranial magnetic stimulation

## Abstract

Supplementary motor area syndrome (SMAS) represents a common neurosurgical sequela. The incidence and time frame of its occurrence have yet to be characterized after surgery for brain tumors. We examined patients suffering from a brain tumor preoperatively, postoperatively, and during follow-up examinations after three months, including fine motor skills testing and transcranial magnetic stimulation (TMS). 13 patients suffering from a tumor in the dorsal part of the superior frontal gyrus underwent preoperative, early postoperative, and 3-month follow-up testing of fine motor skills using the Jebsen-Taylor Hand Function Test (JHFT) and the Nine-Hole Peg Test (NHPT) consisting of 8 subtests for both upper extremities. They completed TMS for cortical motor function mapping. Test completion times (TCTs) were recorded and compared. No patient suffered from neurological deficits before surgery. On postoperative day one, we detected motor deficits in two patients, which remained clinically stable at a 3-month follow-up. Except for page-turning, every subtest indicated a significant worsening of function, reflected by longer TCTs (*p* < 0.05) in the postoperative examinations for the contralateral upper extremity (contralateral to the tumor manifestation). At 3-month follow-up examinations for the contralateral upper extremity, each subtest indicated significant worsening compared to the preoperative status despite improvement to the immediate postoperative level. We also detected significantly longer TCTs (*p* < 0.05) postoperatively in the ipsilateral upper extremity. This study suggests a long-term worsening of fine motor skills even three months after SMA tumor resection, indicating the necessity of targeted physical therapy for these patients.

## Introduction

The supplementary motor area is defined as the posterior part of the superior frontal gyrus [1], part of the premotor cortex, anterior to the precentral gyrus and located on the medial surface of the cortex [2].

This part of the brain is responsible for planning complex movements of the contralateral distal and proximal extremities [3–5]. However, the SMA is also involved in movement planning and performance for the ipsilateral upper and lower extremities [6, 7]. By stimulating the primary motor cortex, the dorsal premotor area, and especially the SMA, Montgomery et al. could record electromyographic activity in the ipsilateral extremities in monkeys [6]. Porro et al. were also able to demonstrate that both the contralateral and the ipsilateral SMA are involved in unilateral finger movements, studying hemodynamic changes in the motor cortex during actual motor performance by using functional magnetic imaging (fMRI) [7].

The SMA is located parasagittal, medial to Brodmann´s area 6 behind the posterior margin of the superior frontal lobe [8].

Anatomically, a series of subdivisions in the SMA region concerning the SMA complex exist. First, it can be divided into the pre-SMA region, anterior to the vertical line through the anterior commissure, and the SMA region posterior to this line [9]. Secondly, it can be divided into three areas or parts: the anterior region, involved in producing language (pre-supplementary motor area); an intermediate location, which regulates complex movements of the upper extremities; and a posterior area, which is involved in regulating movements of the lower extremities [8, 10].

The postoperative supplementary motor area syndrome (SMAS), often followed by the resection of a tumor in the SMA, is characterized by motor deficits, including paresis or plegia, and speech disorders, including hesitancy or mutism [11–14]. According to general clinical assumptions supported by recent literature, SMAS will recover over time and is a transient disorder [11, 12, 14, 15].

This study aimed to investigate the SMAS by examining fine motor skills using the Jebsen-Taylor Hand Function Test (JHFT), the Nine-Hole Peg Test (NHPT) combined with a preoperative mapping of the motor function via navigated transcranial magnetic stimulation (nTMS) to precisely rule out motor cortex involvement in postoperative neurological deterioration in patients suffering from a tumor in the SMA preoperatively, postoperatively (with one week after surgery), and during 3-month follow-up testing.

## Methods and materials

### Ethics

Before each enrolment, written informed consent was signed by each patient. The local ethics committee of our university approved all aspects of the current study (Ethics Committee Registration Number 293/17) by the Declaration of Helsinki.

### Testing fine motor skills

Patients underwent preoperative, early postoperative (within one week after surgery), and 3-month follow-up testing of the fine motor skills using the JHFT and the NHPT. The JHFT consisted of 7 subtests for testing both upper extremities. This test battery is an objective and standardized item to measure fine and gross motor function [16–19]. The subtests of the JHFT consisted of writing first, in which the participant was asked to write the same sentence with both hands, followed by simulated page turning, in which the patient turned pages placed in front of him as fast as possible and lifted small objects, like coins or safety pins, in a can again as quickly as possible. Subsequently, the patients were encouraged to the following modalities: simulated feeding (picking up small objects with a spoon and putting them in a can), stacking checkers (the checkers are supposed to stack on each other), lifting large, light objects, in which the subjects were asked to replace large, light cans, followed by lifting large, heavy objects (replacing and lifting large, heavy cans). Finally, the patients had to solve the NHPT, in which nine sticks had to be placed in nine holes as fast as possible.

While performing all subtests with both upper extremities, the participants’ completion times (TCTs) were recorded preoperatively, postoperatively, and during the three-month follow-up testing and compared afterward between time points and sides.

### Preoperative navigated transcranial magnetic stimulation (nTMS) of the motor cortex

Presurgical navigated transcranial magnetic stimulation (nTMS) induces an electric field within the motor cortex, followed by a neuronal depolarization which results in the development of an action potential. This action potential is transmitted to the muscles and can be measured as a motor-evoked potential (MEP) [20, 21]. We performed nTMS cartography to exclude patients suffering from tumors spreading to the primary motor area with direct motor cortex involvement.

**Fig. 1:**
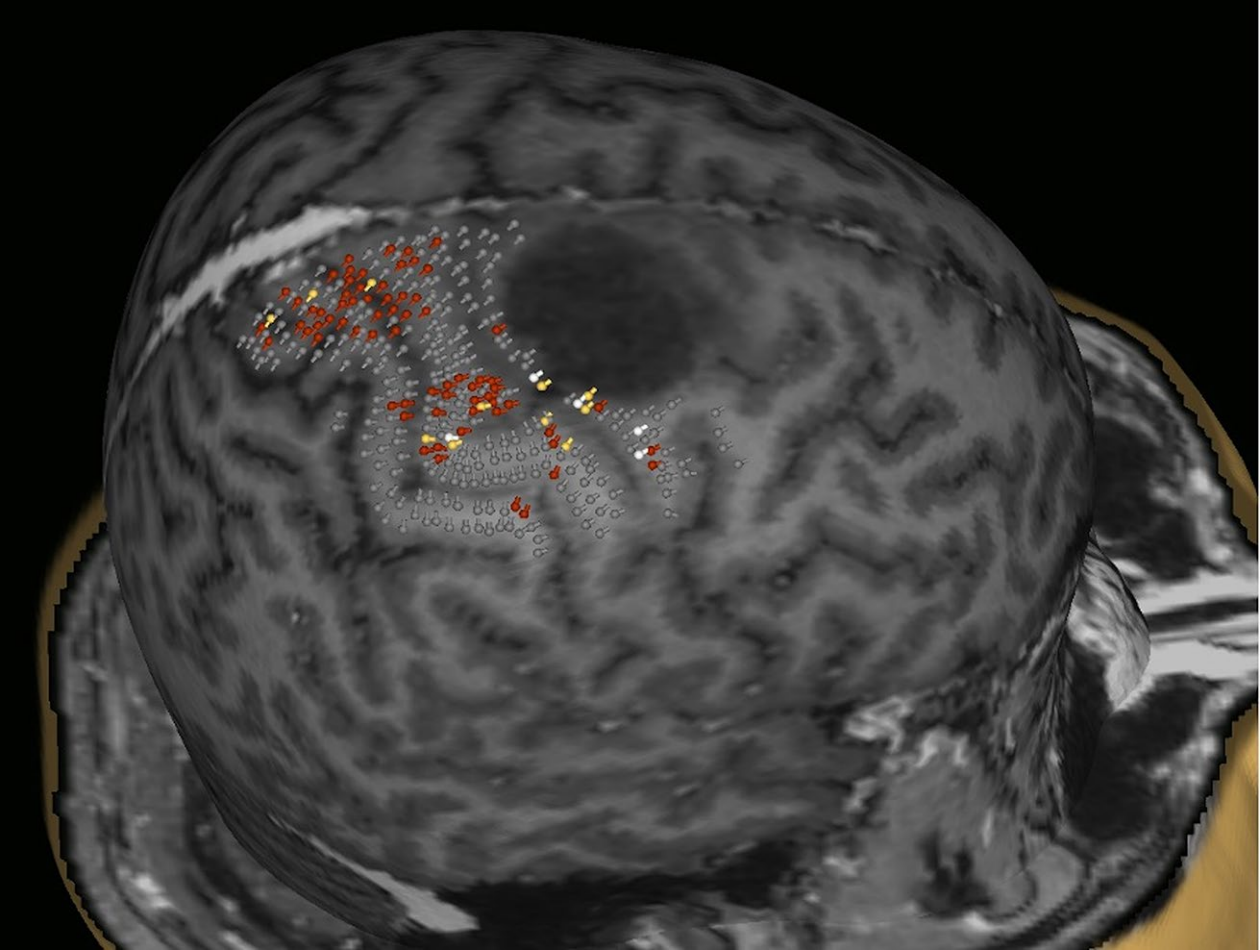
Illustration of navigated Transcranial Magnetic Stimulation (nTMS) data within the neuronavigation. Grey: cortical areas with negative MEP answers, red: positive MEP answers displaying the functional motor cortex of this patient suffering from a tumor in the SMA

For mapping the motor cortex, we used the Nexstim eXimia NBS system, version 3.2 or 4.3 (Nexstim Plc., Helsinki, Finland), in combination with a biphasic figure-of-eight magnetic coil and an integrated infrared tracking system for real-time neuronavigation (Polaris Spectra, Waterloo, Ontario, Canada) [22–24]. The examinations were performed with the preoperative three-dimensional (3D) T1-weighted contrast-enhanced gradient magnetic resonance imaging (MRI) sequences for neuronavigation. All mapping examinations were conducted according to a standardized stimulation protocol by fully trained investigators, as previously reported by [23, 25]. We monitored the motor responses continuously with the integrated electromyography (EMG). The motor responses from the abductor pollicis brevis muscle (APB), abductor digiti minimi muscle (ADM), flexor carpi radialis muscle (FCR), biceps brachii muscle, tibialis anterior muscle and gastrocnemius muscle were recorded. Therefore, we placed electrodes (Neuroline 720, Ambu, Ballerup, Denmark) over the muscle bodies of the limbs contralateral to the brain tumor and a reference electrode over the tendon/bone transition area. Afterward, the resting motor threshold (rMT) was determined, and the mapping was performed using 110% rMT [23]. The mapping examination was started at the hand knob. It was conducted in 3–5 mm steps perpendicular to the sulci until the magnetic stimulation did not elicit any further MEP in any direction (Fig. 1). All cortical spots that produced an MEP were evaluated as positive for the cortical representation of the mapped muscles and exported from the TMS system via the DICOM standard. Afterward, a fiber tracking of the corticospinal tract was created with the exported data. We examined 11 patients detecting their motor-relevant areas via nTMS preoperatively and one postoperatively.

### Statistics

We used PRISM 7 for Mac OS X, Version 7.0b for statistical analysis.

The preoperative, early postoperative, and follow-up TCTs were compared using the Friedmann test for descriptive statistics and the Wilcoxon test. We analyzed the test results in terms of lengthening or other changes of the TCTs followed by surgical resection of a tumor in the SMA over time. Therefore, we compared the preoperative and early postoperative TCTs and the preoperative TCTs versus the detected TCTs during the three-month follow-up examinations. Results are described as mean±standard deviation.

All results are presented as odds ratios (OR) with 95% confidence intervals (CI). The level of significance was 0.05 (two-sided) for each statistical test.

## Results

### Patient population

We performed a single-center, prospective study in which we enrolled 13 patients suffering from a tumor in the dorsal part of the superior frontal gyrus. The median age was 46.6 years. Eight participants were male (61.5%), and five were female (38.5%). All but one patient were right-handed. The surgical procedures in patients considered for this study lasted from January 2015 until January 2017. Histopathological findings postoperatively confirmed four anaplastic oligodendrogliomas, three glioblastomas, three diffuse astrocytomas, two metastases, and one anaplastic astrocytoma. In ten cases, the tumor was in the right hemisphere, and three patients suffered from a left-hemispheric tumor. Additionally, we assessed the motor function preoperatively using the British Medical Research Council (BMRC) scale. Inclusion criteria were age above 18 years, a tumor in the dorsal part of the superior frontal gyrus without involvement of the precentral gyrus, in-house surgical treatment, and no impairment of motor strength in the preoperative examination.

### Writing

Concerning the writing task for the extremity contralateral to the brain tumor, we detected a significant worsening comparing the preoperative (mean 38±22 s) and postoperative (mean 73±64 s) test completion times (TCTs) with a p-value of 0.001 (Table 1). Comparing the preoperative and three-month follow-up test results, we still found significant worsening (p-value 0.0015) with mean TCTs of 68±55 s. One participant couldn´t be tested postoperatively and during the follow-up examination for the contralateral upper extremity due to a paresis concerning all JHFT and the NHPT tasks.

**Table 1:** Results from the test battery of the Jebsen-Taylor Hand Function Test (JHFT) and the Nine-Hole Peg Test (NHPT)

Test battery JHFT	Preoperative TCTs (mean in seconds±SD)	Postoperative TCTs (mean in seconds)	3 months follow-up TCTs (mean in seconds)	p-value preoperative vs. postoperative	p-value postoperative vs. 3 months follow-up
Writing contralateral	38 ± 22	73 ± 64	68 ± 55	0.001	0.0015
Writing ipsilateral	30 ± 17	34 ± 19	33 ± 19	0.0093	0.0173
Simulated page turning contralateral	10 ± 4	17 ± 13	16 ± 10	0.0581	0.0186
Simulated page turning ipsilateral	8 ± 3	10 ± 7	10 ± 6	0.1289	0.0508
Lifting small objects contralateral	9 ± 3	15 ± 9	16 ± 7	0.0156	0.0039
Lifting small objects ipsilateral	8 ± 2	9 ± 2	9 ± 2	0.0273	0.125
Simulated feeding contralateral	11 ± 3	18 ± 10	17 ± 8	0.001	0.0029
Simulated feeding ipsilateral	9 ± 2	10 ± 2	10 ± 1	0.1479	0.1357
Stacking checkers contralateral	8 ± 3	14 ± 11	13 ± 8	0.0078	0.001
Stacking checkers ipsilateral	6 ± 2	8 ± 3	8 ± 2	0.0039	0.0005
Lifting light objects contralateral	6 ± 2	12 ± 12	11 ± 9	0.0078	0.0078
Lifting light objects ipsilateral	6 ± 2	7 ± 3	7 ± 2	0.0215	0.002
Lifting heavy objects contralateral	6 ± 2	10 ± 7	10 ± 4	0.001	0.001
Lifting heavy objects ipsilateral	5 ± 1	7 ± 3	6 ± 2	0.0088	0.001
Nine-Hole Peg Test contralateral	29 ± 14	52 ± 41	51 ± 32	0.0039	0.0015
Nine-Hole Peg Test ipsilateral	23 ± 5	26 ± 8	25 ± 4	0.061	0.1836

Summary of the test completion times (TCTs) and the p-values for the contralateral and the ipsilateral upper extremity performing the Jebsen-Taylor Hand Function Test (JHFT) and the Nine-Hole Peg Test (NHPT) preoperatively, postoperatively and during the three months follow-up examinations

**Fig. 2:**
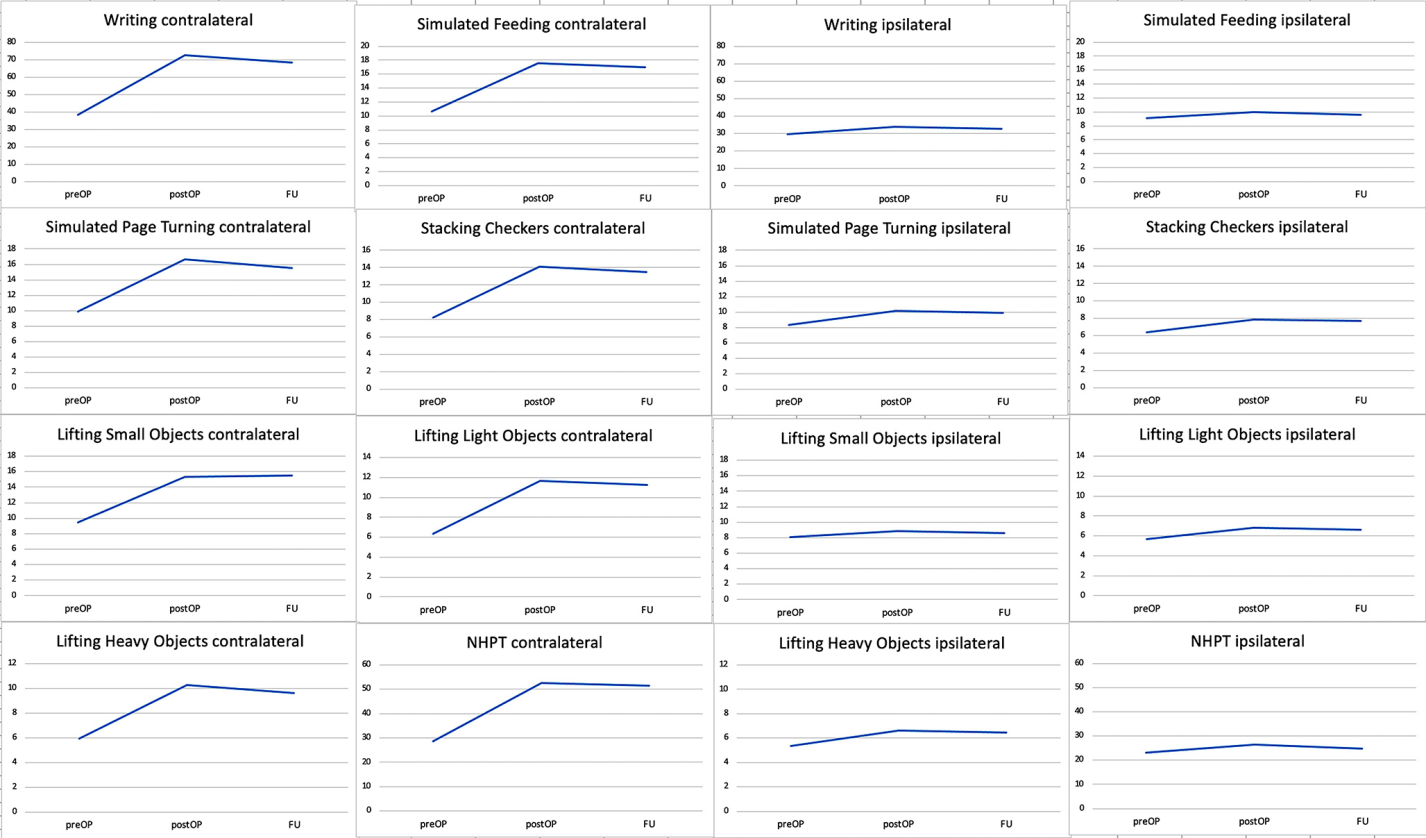
Test completion times before surgery, after surgery, and at follow-up examination for the contralateral and ipsilateral upper extremity

Testing the ipsilateral upper extremity preoperatively (mean 30±17 s), postoperatively (mean 34±19 s), and after the three-month follow-up examination (mean 33±19 s), the TCTs were again significantly longer with p-values of 0.0093 and 0.0173. (Fig. 2)

### Simulated page turning

Simulated page turning for the contralateral upper extremity showed no statistical significance comparing the TCTs preoperatively (mean 10±4 s) and early postoperative (mean 17±13 s) (p-value 0.0581), but significant worsening testing of the preoperative TCTs and the three months follow-up TCTs (mean 16±10 s) with a p-value of 0.0186 (Table 1).

In terms of the ipsilateral upper extremity, we couldn´t detect statistical significance comparing the preoperative (mean 8±3 s), the postoperative (mean 10±7 s) (p-value 0.1289), and the follow-up (mean 10±6 s) (*p* = 0.0508) TCTs. (Fig. 2)

### Lifting small objects

The TCTs were significantly different comparing the preoperative (mean 9±3 s) and the postoperative (mean 15±9 s) results (p-value 0.0156), as well as for the comparison of the preoperative and the follow-up TCTs (mean 16±7 s) (p-value 0.0039) examining the contralateral hand (Table 1).

Only the comparison of the preoperative (mean 8±2 s) and the postoperative (mean 9 ±2 s) TCTs showed a statistically significant worsening for the ipsilateral upper extremity (p-value 0.0273), comparing the preoperative versus the follow-up test results (mean 9±2 s) we detected a p-value of 0.125. (Fig. 2)

### Simulated feeding

The TCTs of the preoperative (mean 11±3 s) versus the postoperative TCTs (mean 18±10 s) showed a statistically significant worsening with a p-value of 0.001 testing the contralateral hand, as well as a statistically significant worsening comparing the preoperative with the three-month follow-up TCTs (mean 17±8 s) (p-value 0.0029) (Table 1). Examining the ipsilateral upper extremity, we could not show any statistically significant worsening in terms of the preoperative (mean 9±2 s), the postoperative (mean 10±2 s) (p-value 0.1479), and the follow-up TCTs (mean 10±1 s) (p-value 0.1357). (Fig. 2)

### Stacking checkers

Concerning the stacking checker’s task, we detected a postoperative significant worsening (mean preoperative 8±3 s, postoperative 14±11 s) of the TCTs with a p-value of 0.0078. Regarding the three-month follow-up examinations, we still observed a significant extension of the TCTs with a mean of 13±8 s completion times (p-value 0.001) (Table 1).

The examination of the ipsilateral upper extremity again showed a statistical deterioration of the TCTs comparing the preoperative (mean 6±2 s) and postoperative (mean 8±3 s) results (p-value 0.0039), as well as for the comparison of the preoperative and follow-up results (mean 8±2 s) (p-value 0.0005). (Fig. 2)

### Lifting light objects

Having a look at the TCTs for lifting light objects with the contralateral hand, we observed a significant deterioration concerning the preoperative (mean 6±2 s) and postoperative (mean 12±12 s) composition, as well as for the comparison of the preoperative and the follow-up examination (mean 11±9 s) with a p-value of 0.0078 each.

Regarding the TCTs of the ipsilateral hand, completion times again were significantly longer (p-value 0.0215 preoperative (mean 6±2 s) vs. postoperative (mean 7±3 s), p-value 0.002 preoperative vs. three months follow-up (mean 7±2 s)) (Fig. 2).

### Lifting heavy objects

Testing the lifting of heavy objects with the contralateral upper extremity, we detected a significant worsening of completion times comparing the preoperative (mean 6±2 s) and the postoperative (mean 10±7 s) results (p-value 0.001), as well as for the comparison of the preoperative and the follow-up (mean 10±4 s) TCTs with a p-value of 0.001.

The TCTs examining the ipsilateral hand also showed a significant worsening for the comparison of the preoperative (mean 5±1 s) and the postoperative (mean 7±3 s) testing (p-value 0.008), as well as for the preoperative vs. the follow-up fine motor skills testing (mean 6±2 s) (p-value 0.001) (Fig. 2).

### Nine-Hole Peg Test

Performing the NHPT, we detected a significant deterioration with a p-value of 0.0039, comparing the preoperative (mean 29±14 s) and the postoperative (mean 52±41 s) TCTs. The completion times of the preoperative vs. the three-month follow-up testing (mean 51±32 s) also showed statistical significance compared to the TCTs (p-value 0.0015) (Table 1).

Examining the ipsilateral hand, we could not show any statistical significance comparing the preoperative - (mean 23±5 s) with the postoperative TCTs (mean 26±8 s) (p-value 0.061), and the preoperative - with the follow-up TCTs (mean 25±4 s) (p-value 0.1836) (Fig. 2).

### Navigated transcranial magnetic stimulation

In total, 11 of the 13 patients underwent a preoperative mapping of the cortical motor function, followed by fiber tracking for the corticospinal tract (Fig. 1). One Patient was examined postoperatively. The mean resting motor threshold (rMT) was 36.6% ± 29%. Two patients who received a motor mapping preoperatively worsened postoperatively concerning the British Medical Research Council scale (BMRC 2/5). One of the patients continued to deteriorate (BMRC 1/5), and the other remained stable (BMRC 2/5) in the 3-month follow-up testing. The other 11 patients showed no deficit postoperatively regarding the BMRC motor function.

### Follow-up

Regarding the contralateral hand, only one patient could not solve the test battery of the JHFT and the NHPT postoperatively and during the three-month follow-up due to postoperative paresis. The median last follow-up concerning the survival rate was 16.2 months (range 39, minimum 3.4 months, maximum 42.4 months). Five patients died during this period.

At three months following surgery, an improvement of just writing (83.3% of all patients) could be detected in the contralateral upper extremity compared to the postoperative state. All the other TCTs from the JHFT were prolonged compared to the postoperative state. Regarding the ipsilateral upper extremity, the writing function recovered after three months (53.8%). The other fine motor skills did not improve and remained impaired.

## Discussion

### Summary

In our study, we identified the worsening of fine motor skills after surgery and at follow-up after the resection of tumors infiltrating the SMA. TCTs including several fine motor skills tests such as writing, lifting light and heavy subjects and JTHF tests were significantly impaired after surgery, and the impairment persisted after three months.

### Permanent supplementary motor area syndrome (SMAS)

A common assumption in neurosurgical practice was that, unlike motor cortex injury, SMAS is transient or doesn´t occur after resection of tumors in the SMA [26–28].

Recently, Palmisciano et al. reviewed the current literature regarding SMAS and the clinical progress after brain tumor resection and showed slightly different results [11]. In his review, 31 studies with 236 patients were included. 94.5% of the tumors in the SMA were gliomas. A gross total resection was performed in 46.3% of all patients, and a complete resection of the SMA was detected in 69.4%. Intraoperative neuromonitoring (direct cortical/subcortical stimulation, motor - or somatosensory evoked potentials) was used in 91.1% of the procedures. The postoperative SMAS occurred within the first 24 h, mostly with motor deficits (97%) and speech disorders (53%). The average duration of the symptoms was 45 days, and 79.9% of the patients improved utterly. 20.3% of the patients, on the other hand, were suffering from permanent symptoms (mostly speech impairment (60.4%) and fine motor disorders (45.8%)). Our results indicate a higher rate of persisting deficits and worsening fine motor skills after 90 days. In comparison, 75% of our patients showed a statistically significant worsening of the fine motor skills in both hands directly postoperative and during the three-month follow-up examinations while performing the JHFT and the NHPT.

At three months following surgery, only an improvement of the write function (83.3% concerning the contralateral hand) could be detected. The other fine motor skills did not improve and mainly remained impaired after 90 days following the resection of a tumor in the SMA. In total, 75% of our patients showed a statistically significant worsening of the fine motor skills in both hands directly postoperative while performing the JHFT and the NHPT. Performing the same tests after three months, 75% of our patients showed significantly worsening their fine motor skills concerning both upper extremities. Analyzing potential influencing factors such as age, sex, tumor side or type of tumor, we did not identify any risk factor significantly associated with longer TCTs after tumor resection. Interestingly, we found an association between the deterioration in performing fine motor skills postoperatively and at follow-up in our left-handed patient (contralateral writing, *p* = 0.006; NHPT *p* = 0.028 at follow-up compared to the preoperative absolute values).

Furthermore, most literature described postoperative motor or fine motor deficits manifesting mainly in the contralateral extremities [4, 5, 10]. Our results underline not only the development of postoperative fine motor skills impairment in the contralateral upper extremity but also in the ipsilateral upper limb of the patients. These neurological deficits were not detected using the British Medical Research Council scale (BMRC) in a routine postoperative examination. They were only seen by having a precise view of fine motor skills using the JHFT and the NHPT. Our results indicate a bilateral postoperative worsening of the fine motor skills after tumor resection in the SMA.

### Preoperative SMA mapping via navigated nTMS

Unfortunately, we did not perform SMA mapping before or after surgery. In terms of using nTMS on the SMA, Schramm et al. conducted a study in 2019 in which nTMS was applied over the SMA in a cohort of 20 healthy subjects and induced fine motor skills impairment, as well as a slowdown of the TCTs [29]. Furthermore, participants accidentally used the contralateral limb for the tests’ completions or could not coordinate the movements during the stimulation.

In 2023, Engelhardt et al. provided a protocol for mapping the SMA via nTMS [30]. nTMS stimulation of the SMA led to a significant reduction of finger taps compared to the baseline (p-value 0.01). Writing, targeting of circles, and line tracing were less accurate than the stimulation of just M1. They concluded that mapping the SMA is feasible but also outlined that although the errors induced in the SMA are not entirely independent of M1, the disruption of the SMA caused functionally distinct errors.

### Risk factors, clinical course, and therapeutic approaches for supplementary motor area syndrome (SMAS)

One of the goals of neurosurgical resection of a tumor in the SMA should be the attempt to minimize the risk of suffering from an SMAS postoperatively.

Russel et al., therefore, listed some risk factors for the development of SMAS followed by the resection of a tumor in the SMA [31]. First, low-grade gliomas in the SMA are associated with a higher incidence of SMAS postoperatively. Secondly, when the extent of resection is limited to the tumor boundaries and the radiographic limitations, the incidence and severity of SMAS may be minimized.

Another risk factor is the proper resection of the SMA posterior to the VCA-line [32].

Further risk of developing an SMAS is trespassing the medial part of the SMA, adjacent cingulate gyrus, and callosal commissural fibers [32–35].

A study from Hatipoglu et al. pointed out the importance of intraoperative monitoring techniques, like direct cortical stimulation, during the resection of a tumor in the SMA or even an awake craniotomy [36].

One possible way to predict the speed of recovery from SMAS postoperatively is diffusion tensor imaging (DTI) tractography [35].

Otten et al. hypothesize that motor functions may return postoperatively as brain motor networks are restored to preoperative conditions by redistributing network functions to other cortical areas and the contralateral hemisphere [37]. This hypothesis is validated by Vassal et al., who detected a significant decrease in inter- but not intrahemispheric connectivity following the resection of the SMA [38]. They found increased interhemispheric connectivity at the three-month follow-up and complete recovery compared to the direct postoperative values. Unfortunately, we did not perform DTI tractography, which presents a limitation of our study.

Another important fact is that the contralateral SMA is essential in restoring function [39–41]. Brain plasticity compensates for ipsilateral function loss due to tumor growth by recruiting the contralateral SMA using interhemispheric connections.

A study about TMS over the SMA in children with Tourette Syndrome pointed out that TMS was even feasible to reduce the severity of the symptoms [42]. A possible treatment for persisting SMAS after tumor resection might be the application of repetitive nTMS over unilateral or bilateral SMA.

Until now, no studies have been published about physiotherapeutic exercises and muscle re-education following SMAS. We consider intensive physiotherapeutic training following surgery in the SMA to be highly important for improving fine motor skill disturbance.

## Limitations

The lack of randomization and the number of patients are significant limitations of the current study and the statistical power.

As the indication for operative resection of the tumor in the SMA was applied in every case, a non-surgically treated control group was missing.

Another significant limitation is the possible habituation of the JHFT and the NHPT, as it was performed six times in total over the period of 3 months (every test was performed with the right and the left hand). We observed a deterioration postoperatively and at follow-up, and the habituation may even underestimate the clinical worsening.

Moreover, we included tumors in both hemispheres, regardless of the hemispheric dominance or handedness of the patient. On the other hand, both upper extremities (except one patient who could not perform the JHFT and the NHPT with the contralateral limb postoperatively and during the three-month follow-up testing) were examined during each test round. In our manuscript, we described mean TCTs but, in fact, also observed very individual differences and stable results over time in a few patients.

Our study did not include tractography, which could depict disruption of the frontal aslant tract [43, 44].

## Conclusion

Our study detected an SMAS with fine motor skills worsening in all patients undergoing resection of SMA tumors. We ruled out the involvement of the motor cortex by performing preoperative nTMS. We conducted extensive neurological testing, applying various tests for fine motor skills, showing a persistent but improving worsening at the three-month follow-up.

## Data Availability

The data supporting this study’s findings are available on request from the corresponding author (Vicki M. Butenschoen).

## Abbreviations

3D: Three-dimensional
ADM: Abductor digiti minimi muscle
APB: Abductor pollicis brevis muscle
BMRC: British Medical Research Council
CI: Confidence interval
EMG: Electromyography
FCR: Flexor carpiradialis muscle
fMRI: Functional Magnetic Resonance Imaging
JHFT: Jebsen-Taylor Hand Function Test
MEP: Motor evoked potential
MI: Primary motor cortex
MRI: Magnetic Resonance Imaging
NHPT: Nine-Hole Peg Test
nTMS: Navigated transcranial magnetic stimulation
OR: Odds ratio
pre SMA: Pre-supplementary motor area
rMT: Resting motor threshold
SMA: Supplementary motor area
SMAS: Supplementary motor area syndrome
TCT: Test completion time
